# 4309 Ascertaining the Medical Needs of Galveston County

**DOI:** 10.1017/cts.2020.255

**Published:** 2020-07-29

**Authors:** Sharon Croisant, Krista Bohn, John Prochaska

**Affiliations:** 1 The University of Texas Medical Branch

## Abstract

OBJECTIVES/GOALS: Data were collected or abstracted from a wide variety of sources related to health and health care needs to determine the current health status of the Galveston community including:

- Demographics
- Social Determinants of Health
- Health Care Access and Insurance Status
- Poverty and Socio-Economic Indicators Impacting Health
- Health Behaviors
- Chronic Disease
- Communicable Disease
- Birth Outcomes
- Mortality
- Cancer
- Data on Services Provided at UTMB
- Data on Services Provided through the Galveston County Health District
- Data on Services Provided through the St. Vincent’s House Clinics, student-led clinics operated at a local non-profit organization
- Previous Galveston County Community Health Needs Assessment
- Identifying Gaps in Services
- Prevention Quality Indicator Data

METHODS/STUDY POPULATION: In addition to collection and analysis of secondary data, we also interviewed key stakeholders to solicit their input and recommendations. We met with leadership from St. Vincent’s House regarding current services provided, perceived issues and concerns, and needs for improvements. We met with leaders from UTMB’s academic enterprise to discuss the operation of our current student-led clinics as well as ways in which clinical practice experiences might be expanded and included more formally in the student curricula should the clinical capacity of St. Vincent’s House also be significantly expanded. This would increase the number of services that could be offered at St. Vincent’s and greatly increase the capacity for enrolling patients without relying on faculty volunteers to staff the clinics. We also met with UTMB leaders in a position to provide insight to issues that bridge the UTMB practice arena and public health and with Community Health leaders from the Galveston County Health District and Teen Health Clinics. Information Services leadership and Institute for Translational Science informatics faculty and staff were instrumental in determining what data could be abstracted from the Electronic Medical Record (without patient identifiers) to determine the specific need for services at St. Vincent’s. RESULTS/ANTICIPATED RESULTS: The City of Galveston has a population just under 50,000. Since 2010, the proportion of elderly has increased, and the proportion of families with younger children has decreased. Poverty is high at 22.3% for all people, and especially high for children at 32.1%. Poverty disproportionately affects racial and ethnic minorities, with 36.5% of the Black population living below the poverty level, compared to 25.5% Hispanic, 30.5% Asian, and 14.7% White. Home ownership is decreasing, and median rent costs have sharply increased. The percentage without health insurance is considerable, driven by educational attainment, age, and race. In 2017, >40% of renters spent more than 35% of their income on housing. Upwards of 2,650 reported not having access to a vehicle for transportation. While residents of Galveston County as a whole are less impoverished, those that are impoverished share marked similarities. Lower educational attainment, in particular failure to complete high school or obtain a college degree, are correlated with race. Lower educational attainment then is highly predictive of poverty and low income. The income inequality ratio, i.e., the greater division between the top and bottom ends of the income spectrum in Galveston County is higher than in Texas or the nation and has increased every year but one since 2010. Issues of concern for Galveston County include obesity, Type II diabetes, and disability. These are exacerbated by built and social environment issues such as food insecurity, limited access to healthy foods, and food deserts in some neighborhoods. Pre-term birth rates are higher in Galveston than in the state or nation, and approximately 40% of women do not receive prenatal care until the 2^nd^ or 3^rd^ trimester or receive no prenatal care at all. 8.4% of births are low-birth weight. Marked disparities by race and ethnicity exist for each of these indicators. Age-adjusted death rates for all-cause mortality are higher in Galveston County than they are in Texas or the United States. Perhaps of most concern are the rates of death from septicemia, which are nearly triple that of the U.S. and nearly double that of the state, and cancer. Cancer incidence is not particularly remarkable, however, cancer age-adjusted mortality rates for many specific cancers well exceed state rates. DISCUSSION/SIGNIFICANCE OF IMPACT: With a clearer picture of the medical and other needs impacting health or health care access for our community, all stakeholders and experts can provide more detailed recommendations about prioritizing care and especially, preventive care—much of which could conceivably be provided in St. Vincent’s House clinics. Opportunities exist for enhanced practice and education opportunities for UTMB students from all schools. Preventive Care and Population Health practices can be brought to bear in novel practice settings that could serve as models for provision of integrated services. Social and other services provided by non-profit organizations can be coordinated and streamlined. It is our hope that the considerable data presented herein will enable stakeholders to begin to prioritize issues and to make some evidence-based decisions about the next steps in this process. Throughout the interview and data collection process, all stakeholders have expressed both enthusiasm and hope at the prospect of re-visioning how they can contribute to a process that will improve how we as a community care for our most vulnerable members. CONFLICT OF INTEREST DESCRIPTION: The authors have no conflicts of interest to disclose.

